# Identification of citrullinated α-enolase as a candidate autoantigen in rheumatoid arthritis

**DOI:** 10.1186/ar1845

**Published:** 2005-10-19

**Authors:** Andrew Kinloch, Verena Tatzer, Robin Wait, David Peston, Karin Lundberg, Phillipe Donatien, David Moyes, Peter C Taylor, Patrick J Venables

**Affiliations:** 1 Kennedy Institute of Rheumatology, Imperial College London, Charing Cross Hospital Campus, 1 Aspenlea Road, London W6 8LH, UK

## Abstract

Antibodies against citrullinated proteins are highly specific for rheumatoid arthritis (RA), but little is understood about their citrullinated target antigens. We have detected a candidate citrullinated protein by immunoblotting lysates of monocytic and granulocytic HL-60 cells treated with peptidylarginine deiminase. In an initial screen of serum samples from four patients with RA and one control, a protein of molecular mass 47 kDa from monocytic HL-60s reacted with sera from the patients, but not with the serum from the control. Only the citrullinated form of the protein was recognised. The antigen was identified by tandem mass spectrometry as α-enolase, and the positions of nine citrulline residues in the sequence were determined. Serum samples from 52 patients with RA and 40 healthy controls were tested for presence of antibodies against citrullinated and non-citrullinated α-enolase by immunoblotting of the purified antigens. Twenty-four sera from patients with RA (46%) reacted with citrullinated α-enolase, of which seven (13%) also recognised the non-citrullinated protein. Six samples from the controls (15%) reacted with both forms. α-Enolase was detected in the RA joint, where it co-localised with citrullinated proteins. The presence of antibody together with expression of antigen within the joint implicates citrullinated α-enolase as a candidate autoantigen that could drive the chronic inflammatory response in RA.

## Introduction

Rheumatoid arthritis (RA) is a common and disabling disease affecting about 1% of the population [1]. Unlike most other autoimmune rheumatic diseases, the dominant autoantigens are unknown. Because rheumatoid factors are present in up to 75% of patients with RA, it has been suggested that immunoglobulin G is the antigen. However, rheumatoid factors are also present in patients with other diseases and in up to 5% of healthy individuals [2]. Other antibodies are also present in sera from patients with RA, including antiperinuclear factor [3] and antikeratin antibody [4]. Because both antiperinuclear factor and antikeratin antibody react with human filaggrin and related proteins [5] they were collectively designated 'anti-filaggrin antibodies'. It was subsequently reported that binding of anti-filaggrin antibody epitopes is dependent on the presence of citrulline, an amino acid derived from arginine as a result of a post-translational modification catalysed by the enzyme peptidylarginine deiminase (PAD) [6,7].

These findings have been exploited in anti-cyclic citrullinated peptide (anti-CCP) assays, which are more sensitive (80%) and specific (97%) for RA than rheumatoid factors are [8]. Anti-CCPs may occur early in disease [9], or even before clinical manifestations [10]. Anti-CCP positivity also predicts a more aggressive form of RA [11,12]. Anti-filaggrin antibodies have been found at higher concentrations in synovial membrane than in synovial fluid and peripheral blood [13] from patients with RA. However, filaggrin is notably absent from the RA joint [8]. This suggested that there might be other citrullinated proteins in the joint driving the immune response. Citrullinated fibrin is a candidate because it is present in interstitial deposits in the synovial membrane [13] and is recognised by anti-citrullinated-filaggrin antibodies. Endogenous citrullination of fibrin has also been demonstrated in murine models of arthritis [14]. However, immunisation of mice with citrullinated fibrinogen did not induce arthritis [15,16]. Another candidate is citrullinated vimentin, now known to be identical to the Sa antigen [17,18], the presence of which has been demonstrated in synovial membrane [19].

It is not known whether citrullinated vimentin and fibrin are just two of multiple citrullinated autoantigens in RA, or whether there is a dominant autoantigen that has yet to be described. The premise of the current study is that, if there were such a candidate, it is likely to be present in myeloid cells, the dominant cell type in the rheumatoid joint. We therefore studied the promyelocytic HL-60 cell line, which can readily be differentiated into cells with a monocytic or granulocytic phenotype that also express PAD [20]. Untreated and citrullinated lysates of HL-60s were probed with an initial screening panel of serum from patients with RA, to identify reactive polypeptides. These were then partly purified and identified by tandem mass spectrometry. This approach has enabled us to propose citrullinated α-enolase as a novel candidate autoantigen for RA.

## Materials and methods

### Patient samples

Serum was obtained with informed consent from 52 patients with RA attending the Rheumatology Clinic, Charing Cross Hospital, London. All met the classification criteria for RA [21]. Control serum samples were obtained from healthy volunteers. Multiple synovial biopsies were taken under direct vision from each of three predetermined sites within the knee joint during arthroscopic examination in eight patients with RA and four with osteoarthritis. Informed consent was obtained from each patient before arthroscopy. All biopsies taken during a single examination were fixed for 24 hours in 10% neutral buffered formalin and then processed into paraffin wax. Ethical approval was granted by the Riverside Research Ethics Committee and the Hammersmith NHS Trust Research Ethics Committee.

### Isolation of RA synovial cells

Synovial cells were isolated from synovium that had been surgically removed from three patients, undergoing total knee, hip or elbow replacement. After the removal of fat, synovium was cut into small pieces in complete medium (RPMI 1640, 10% fetal calf serum, 1% penicillin and streptomycin) in a plastic tissue culture dish. The tissue was drained in a sieve and scraped into a beaker containing 20 ml of complete medium, 100 μg of collagenase A and 3 μg of DNase A, mixed thoroughly and incubated for up to 90 minutes at 37°C until 'stringy'. The mixture was shaken vigorously and diluted with complete medium to a final volume of 50 ml. Synovial cells were pelleted by centrifugation (200 *g *for 10 minutes at 24°C).

### Culture and differentiation of HL-60 cells

HL60 cells were cultured in complete medium and passaged every third day. For differentiation to PAD-expressing monocytes or granulocytes, 3 × 10^5 ^cells/ml were incubated for 3 days with either 100 nM 1α,25-dihydroxyvitamin D_3 _(Wako Chemicals, Neuss, Germany) or 1 μM *trans*-retinoic acid (Sigma, Poole, UK) [20].

### Preparation of whole-cell lysates and subcellular fractionation

Cells were lysed at 2.5 × 10^6 ^cells per 150 μl of lysis buffer (50 mM Tris-HCl, pH 8.0, 150 mM NaCl, 0.1% SDS, 1% Nonidet P40, 100 mg/ml aprotinin). Protein concentrations were measured by the Bio-Rad DC Protein assay (Bio-Rad, Hercules, CA, USA) and diluted with PBS. Subcellular fractionation was performed by resuspending PBS-washed cells in lysis buffer (10 mM Tris-HCl, pH 7.5, 1 mM potassium acetate, 1.5 mM magnesium acetate, 2 mM dithiothreitol, 1 mM phenylmethylsulphonyl fluoride, 10 μg/ml aprotinin, 1 μg/ml leupeptin, 10 μg/ml pepstatin), incubating on ice for 30 minutes and disrupting with a Dounce homogeniser. Homogenates were centrifuged (500 *g *for 10 minutes) to pellet the nuclear fraction, which was washed, disrupted by sonication and solubilised in 0.5% Nonidet P40. The supernatant from the nuclear fractionation was centrifuged at 100,000 *g*, giving a membrane-rich pellet (P100) and a cytosolic supernatant (S100).

### Deimination of proteins *in vitro*

Deimination was performed as described previously [7]. In brief, whole-cell lysates and subcellular fractions were diluted to a concentration of 0.86 mg protein/ml in PAD buffer (0.1 M Tris-HCl, pH 7.4, 10 mM CaCl_2_, 5 mM dithiothreitol, 1 mM phenylmethylsulphonyl fluoride, 10 μg/ml aprotinin, 1 μg/ml leupeptin, 10 μg/ml pepstatin) and were deiminated *in vitro *with rabbit muscle PAD (7 U/mg of protein; Sigma) for 2 hours at 50°C. The reaction was stopped by boiling in Laemmli buffer for 10 minutes. Non-neuronal α-enolase (Hytest, Turku, Finland) was deiminated in the same buffer at a concentration of 0.365 mg/ml. All samples were stored at -20°C until use.

### Immunoblotting

Whole-cell lysates were separated on 10% NuPAGE Bis-TrisGels (Invitrogen, Paisley, Renfrewshire, UK), transferred to nitrocellulose membranes, blocked with 5% non-fat milk in PBS/0.1% Tween, and probed with human serum diluted 100-fold with the blocking solution. Goat anti-α-enolase antibody (Santa Cruz Biotechnology, Santa Cruz, CA, USA) was used at a dilution of 1:100. Membranes were washed three times for 15 minutes with PBS/0.1% Tween and incubated with peroxidase-conjugated secondary antibody (Jackson Immuno Research, West Grove, PA, USA), anti-human (recognising immunoglobulin G, immunoglobulin M and immunoglobulin A) and rabbit anti-goat respectively. After a further wash, membranes were developed with the use of enhanced chemiluminescence (Amersham Biosciences, Little Chalfont, Buckinghamshire, UK) in accordance with the manufacturer's instructions. Deiminated proteins were identified with an anti-citrulline (modified) detection kit (catalogue no. 07–-390; Upstate, Lake Placid, NY, USA). The presence of antibodies against citrullinated and non-citrullinated antigens (1.92 μg per well) was established by blotting with serum at a dilution of 1:40.

### Two-dimensional gel electrophoresis

*In vitro *deiminated S100 fractions of 1α,25-dihdroxyvitamin D_3_-differentiated HL-60 cells were desalted with spin desalting columns (Pierce, Northumberland, UK) and were dissolved in 2D lysis buffer (9.5 M urea, 1% (w/v) dithiothreitol, 2% CHAPS and 0.5% carrier ampholyte (Amersham Biosciences) supplemented with proteinase inhibitors. The samples were loaded by in-gel rehydration into linear pH 3 to 10 immobilised pH gradient dry strips 13 cm long (Amersham Biosciences). Isoelectric focusing was performed with a Multiphor II flatbed electrophoresis system (Amersham Biosciences) at 300 V for 1 minute, then ramped to 3,500 V for 1.5 hours and maintained at 3,500 V for 3.5 hours. Before separation in the second dimension, disulphide bonds were reduced by incubation with 65 mM dithiothreitol (15 minutes in 2% SDS, 6 M Urea, 30% v/v glycerol and 150 mM Tris-HCl, pH 8.8). Free thiol groups were alkylated by treatment with 260 mM iodoacetamide for 15 minutes. The strips were transferred to a 10% polyacrylamide gel and run at 8 mA. Gels were fixed and silver stained with a protocol compatible with mass spectrometry [22].

### Mass spectrometry

In-gel digestion with trypsin was performed with an Investigator Progest robotic digestion system (Genomic Solutions, Huntington, UK) as described previously [23]. Tandem electrospray mass spectra were recorded with a Q-Tof hybrid quadrupole/orthogonal acceleration time-of-flight spectrometer (Micromass, Manchester, UK) interfaced to a Micromass CapLC chromatograph. Samples were dissolved in 0.1% aqueous formic acid, and introduced into the spectrometer by means of a Pepmap C_18 _column (300 μm × 0.5 cm; LC Packings, Amsterdam, The Netherlands), and were eluted with an acetonitrile/0.1% formic acid gradient (5% to 70% acetonitrile over 20 minutes).

The capillary voltage was set to 3,500 V, and data-dependent tandem mass spectrometry acquisitions were performed on precursors with charge states of 2, 3 or 4 over a survey mass range of 400 to 1,300. Proteins were identified by correlation of uninterpreted tandem mass spectra to entries in SwissProt/TrEMBL, using ProteinLynx Global Server (Version 1.1; Micromass) [24]. The database was created by merging the FASTA format files of SwissProt, TrEMBL and their associated splice variants. No taxonomic, mass or pI constraints were applied. One missed cleavage per peptide was allowed, and the fragment ion mass tolerance window was set to 100 p.p.m. All matching spectra were reviewed by an expert, and citrullinated residues were localised by manual interpretation of sequence-specific fragment ions with the MassLynx program PepSeq (Micromass).

### Slide preparation and immunohistochemistry

Synovial tissue biopsies were processed into paraffin wax by fixation in 10% neutral buffered formalin for 24 hours. The tissue was then progressively dehydrated by passage through a series of graded alcohols and xylene. The samples were mounted on silane-treated slides, which were incubated for 10 minutes with 2% hydrogen peroxide/98% methanol, blocked for 10 minutes in horse serum and then incubated for 60 minutes with anti-α-enolase antibody diluted 1:400. Citrullinated proteins were detected with the anti-modified citrullinated protein kit (Upstate). Unmodified sections were used as controls. The slides were washed in TBS and incubated for 30 minutes with either biotinylated horse anti-goat (for enolase) or biotinylated pig anti-rabbit (for citrulline) antibodies, at a concentration of 1:400 and washed again before incubation for 30 minutes with avidin-biotin-HRP (PK6100; Vector Biolabs) at a 1:100 concentration and staining for 5 minutes with diaminobenzidine (SK4100; Vector Biolabs). Finally the slides were counterstained for 1 minute with haematoxylin, washed in tap water, dehydrated, cleared and mounted.

## Results

### Identification of a 47 kDa citrullinated protein as a target for antibodies in sera from patients with RA

**Figure 1:**
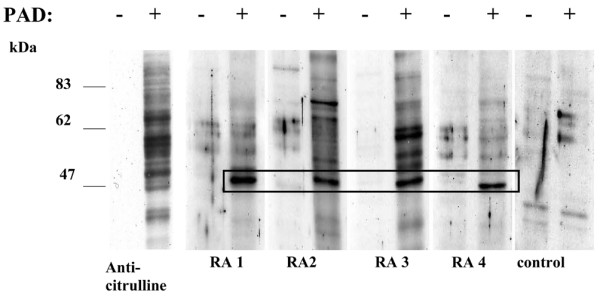
Screen to identify undeiminated and deiminated proteins reacting with RA and non-RA serum samples. Proteins from HL60 lysates incubated (+) or without (-) peptidylarginine deiminase (PAD) blotted with an antibody specific for modified citrulline residues (anti-citrulline) and a screening panel of rheumatoid arthritis (RA1 to RA4) and non-RA (control) serum. The rectangular box indicates a citrullinated protein reacting strongly with each of the RA serum samples but not the control.

Each of four serum samples from patients with RA, but not the control serum, reacted strongly with a band with an apparent mass of 47 kDa (boxed in Figure 1) in the PAD-treated lysates of HL-60 cells. No reaction at 47 kDa was observed with non-deiminated lysates. Reactivity at 47 kDa was strongest with HL-60 cells that had been differentiated to monocytes, although a similar polypeptide was also seen in lysates from cells with the granulocyte phenotype (data not shown). Endogenous citrullination in the HL-60 cells was undetectable with the antibody against modified citrulline, but after treatment with PAD *in vitro*, abundant citrullinated polypeptides were observed. The RA sera, particularly RA1 and RA4, seemed to be relatively selective for the 47 kDa polypeptide, with only four to six additional bands identifiable in each blot. Serum from RA2 and RA3 showed more diffuse reactivity, although a 47 kDa polypeptide predominated. This suggested that, among the numerous potential antigens generated by citrullination of proteins in cells of monocytic phenotype, there was apparent selectivity among our four screening sera for one citrullinated polypeptide migrating at 47 kDa. We therefore performed further experiments to identify this protein.

**Figure 2:**
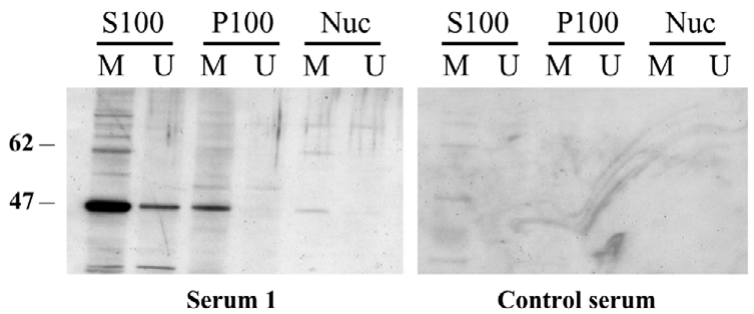
Intracellular expression of immunogenic citrullinated 47 kDa protein. Presence of citrullinated 47 kDa protein reactive with rheumatoid arthritis serum 1 in different subcellular fractions of undifferentiated HL60s (U) and HL60 monocytes (M) (S100, cytosolic; P100, membrane; Nuc, nuclear) showing enrichment in the S100 (cytosolic) fraction.

Nuclear, cytosolic and membrane fractions were prepared from HL-60 cells by differential centrifugation, and were deiminated with PAD as before. Immunoblotting with one of the RA sera showed that the 47 kDa antigen was enriched in the cytosolic (S100) fraction (Figure 2).

### Identification of the 47 kDa autoantigen as citrullinated α-enolase

**Figure 3:**
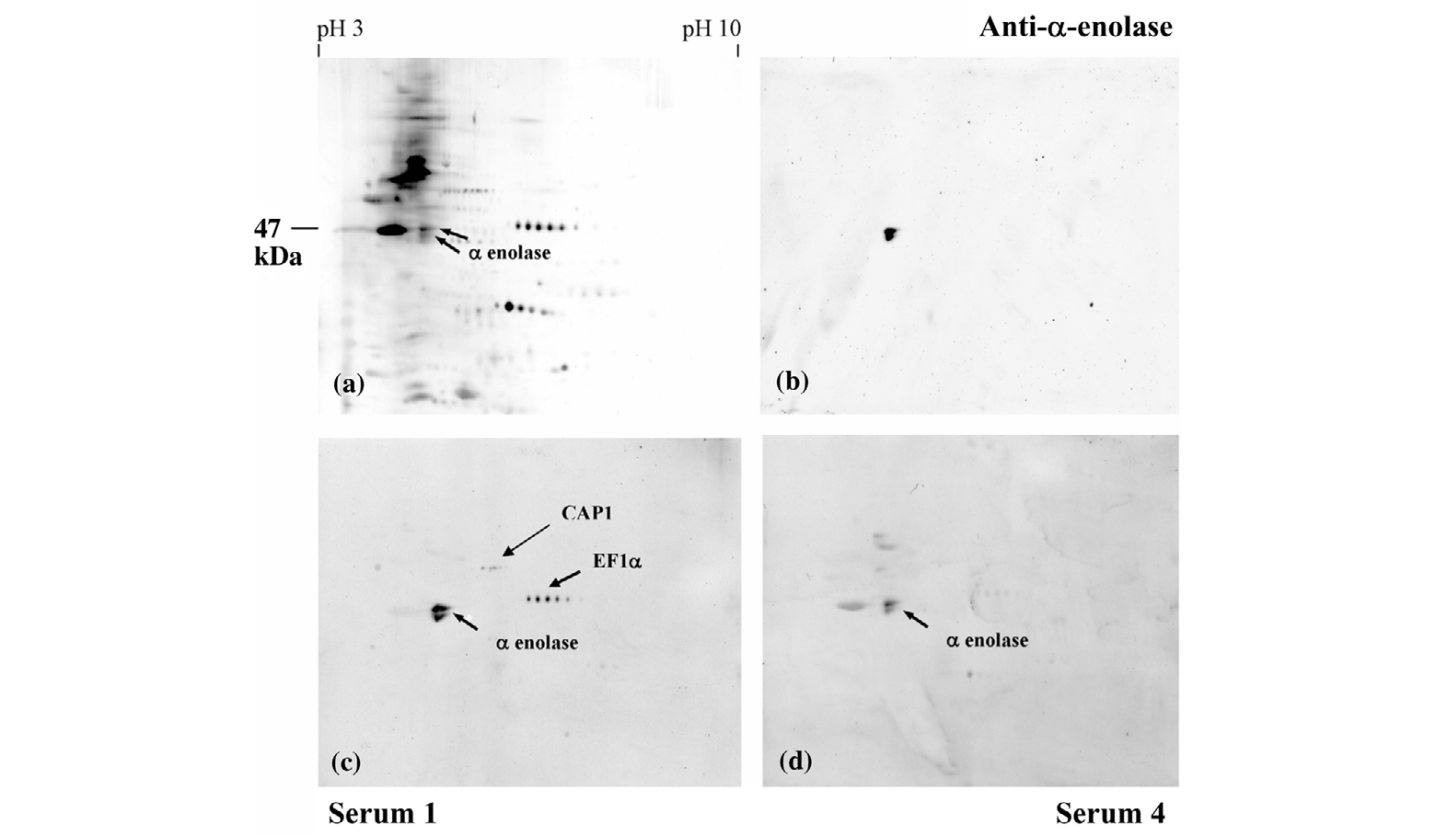
Characterisation of the 47 kDa protein by two-dimensional electrophoresis. Proteins in the 47 kDa rich monocytic S100 fraction were separated by two-dimensional electrophoresis according to charge (*x*-axis) and molecular mass (*y*-axis). **(a) **The full complement of proteins was observed by silver staining. **(c,d) **Proteins reacting with rheumatoid arthritis serum samples 1 (c) and 4 (d) were highlighted by immunoblotting. **(b) **The highly reactive 47 kDa protein was confirmed as α-enolase by immunoblotting with the goat anti-α-enolase antibody. CAP1, adenyl cyclase-associated protein 1; EF1α, elongation factor 1α.

The deiminated S100 fraction was separated by one-dimensional SDS-PAGE and stained with Coomassie blue; the putative band recognised by sera from patients with RA was excised, digested with trypsin and analysed by tandem mass spectrometry. Fourteen peptides were sequenced (Table 1), all of which mapped onto α-enolase (SwissProt accession number P06733). In total 242 residues of non-redundant amino acid sequence were obtained, corresponding to 56% coverage. To confirm that the stained band co-localised with the protein recognised by sera from patients with RA, the deiminated S100 fraction was separated by two-dimensional electrophoresis, blotted onto nitrocellulose and probed with serum samples RA1 and RA4. Both recognised a doublet of spots that matched a feature on the silver-stained gel of apparent molecular mass 47 kDa, and with a pI of 5 (Figure 3). These spots were excised and identified by mass spectrometry as α-enolase. When the membranes were re-probed with an antibody specific for the carboxy-terminal region of α-enolase, we observed a similar pattern to that obtained with serum from patients with RA, confirming the identity of the autoantigen.

Conversion of arginine to citrulline results in a mass increase of 0.984 Da and a loss of the positive charge from the side chain, resulting in a significant acidic shift in the two-dimensional electrophoretic migration. Moreover, the pattern of peptides obtained on tryptic digestion will be altered, because the modified residues are refractory to trypsinolysis and yield peptides containing internal citrulline, rather than carboxy-terminal arginine (Tables 1 and 2). Six peptides containing internal citrulline residues were sequenced, which enabled the localisation of nine sites of modification (Table 1). None of these peptides were present in tryptic digests of unmodified α-enolase (Table 2). The pI determined by two-dimensional electrophoresis was also consistent with this extensive citrullination, being about 5.0.

Other antigens, recognised more sporadically by sera from patients with RA (Figure 3), were also characterised by mass spectrometry. They included elongation factor 1α (SwissProt accession number P68104) and adenyl cyclase-associated protein 1 (SwissProt accession number Q01518), both of which were shown to be citrullinated.

### Higher prevalence of antibodies against citrullinated α-enolase than against native α-enolase in serum from patients with RA

**Figure 4:**
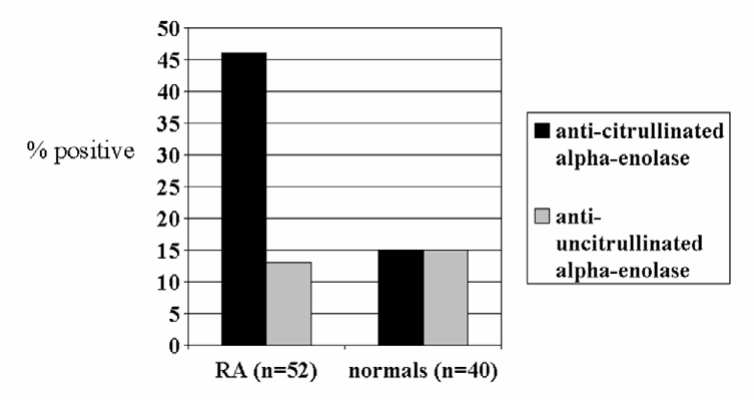
Prevalence of antibodies against deiminated and undeiminated α-enolase in RA patients and healthy controls. Immunoblotting of *in vitro *citrullinated and untreated α-enolase with serum samples from patients with rheumatoid arthritis (RA) and normal controls, showing that about half of the serum samples from the RA group contain antibodies with selectivity for citrullinated α-enolase.

Twenty-four of the RA serum samples (46%) reacted with the citrullinated α-enolase, seven of which (13%) also reacted with the non-citrullinated form of the protein. Six of the controls (15%) reacted with both (Figure 4). All of the 17 RA samples that reacted only with the citrullinated form of α-enolase were positive for anti-CCP2 (data not shown).

### α-Enolase is abundant in synovium from patients with RA

**Figure 5:**
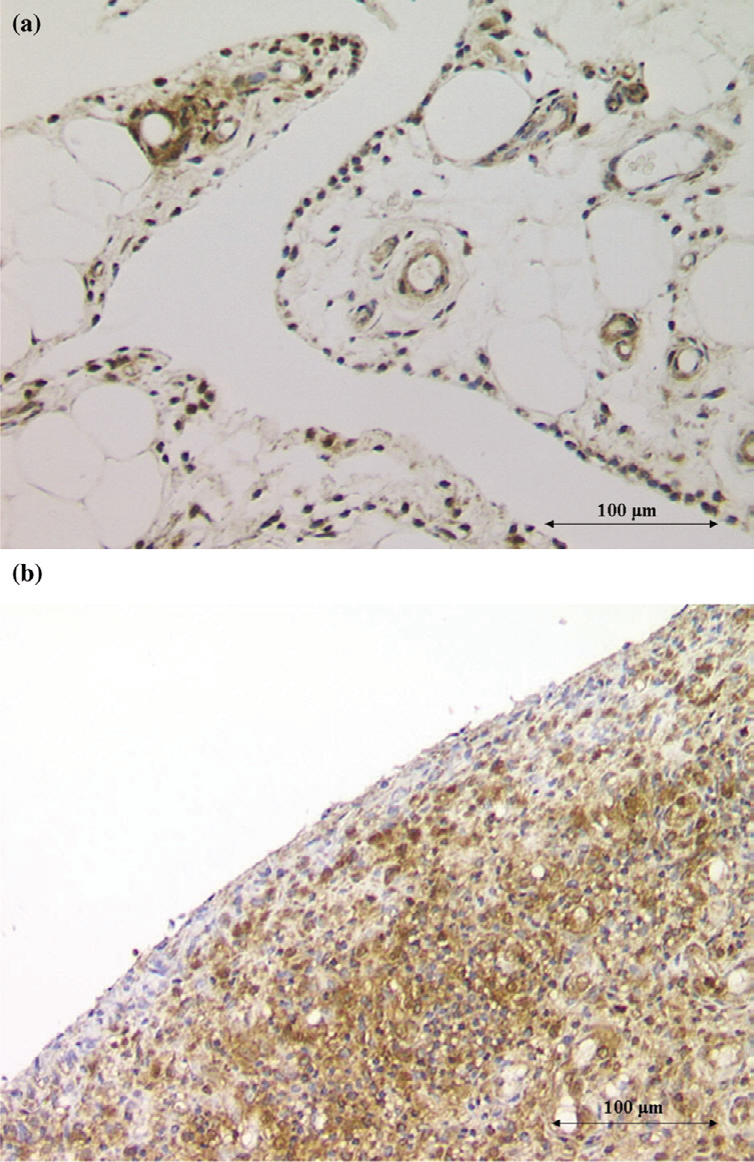
Localisation of α-enolase in synovial membranes. Immunohistochemistry of biopsy sections from patients with osteoarthritis **(a) **and rheumatoid arthritis (RA) **(b) **with the goat anti-α-enolase antibody showing expression of α-enolase (stained brown) in cells in the subsynovium of the patient with RA and in endothelial cells in the patient with osteoarthritis. Cell nuclei are counterstained blue.

**Figure 6:**
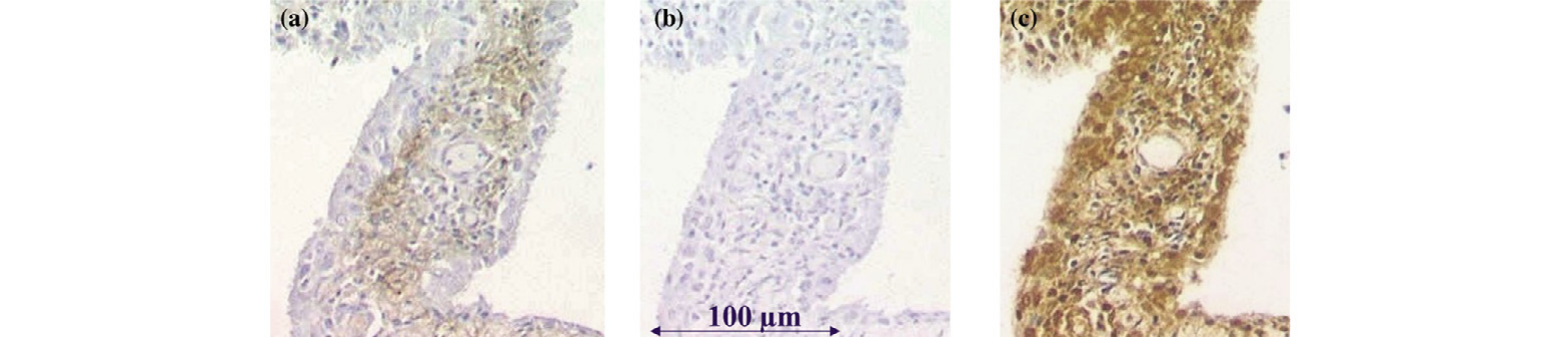
Localisation of citrullinated proteins in synovial membranes. **(a) **Immunostaining of citrullinated proteins by the anti-modified citrulline kit was mainly confined to the subsynovium. **(b) **No staining was visible on the control. **(c) **Staining produced by the anti-α-enolase antibody on an adjacent section was much stronger, and included the subsynovial cells which also stained for citrullinated antigens. Original magnification × 20 in all cases.

**Figure 7:**
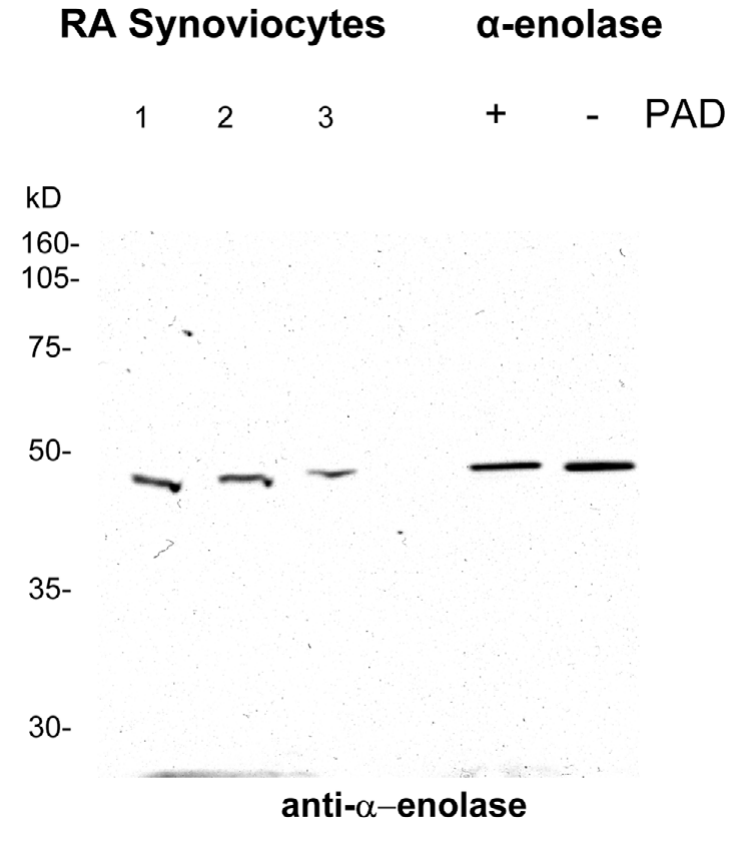
Presence of α-enolase in synovial cells from patients with rheumatoid arthritis (RA). Immunoblotting of lysates from RA synovial cells with anti-α-enolase (+PAD, deiminated α-enolase; – PAD, undeiminated α-enolase) showing a 47 kDa protein in synoviocytes, from three patients with RA, reacting with the goat anti-α-enolase antibody, which co-migrates with purified α-enolase.

Immunohistochemical analysis of inflamed synovial sections showed that all eight RA and four osteoarthritis samples examined expressed α-enolase. Expression in RA sections was greatest in the more hyperplastic subsynovial layer (Figure 5b). In the osteoarthritis sections, α-enolase staining was predominantly localised in vascular endothelial cells (Figure 5a). The antibody against modified citrulline (Figure 6a) indicated that citrullinated proteins were present in the region that stained positively for enolase, although the intensity of the staining indicated that levels of citrullination were relatively low. Immunoblotting of synovial cell lysates from three patients with RA demonstrated that a band co-migrating with purified α-enolase reacted with the anti-α-enolase antibody (Figure 7).

## Discussion

In this study we characterised citrullinated α-enolase as a dominant antigen in citrullinated lysates of differentiated HL-60 cells targeted by a screening panel of serum from patients with RA. The identity of the antigen was established by mass spectrometry, and the sites of nine citrulline residues within the protein were determined by tandem mass spectrometry. Further confirmation was obtained by two-dimensional electrophoresis and Western blotting with a specific anti-enolase antibody. With the use of purified protein, 46% of a larger panel of sera from patients with RA reacted with citrullinated α-enolase by immunoblotting. This suggests that citrullinated α-enolase is at least as immunodominant as citrullinated filaggrin or citrullinated vimentin, because, by immunoblotting, the frequency of antibodies against citrullinated filaggrin has been reported as 41 to 58% [25-28] and against citrullinated vimentin as 22 to 40% [19,29,30]. Improved sensitivity and specificity of RA diagnosis may well be obtained by testing RA sera with peptides derived from citrullinated epitopes of α-enolase, as has been demonstrated for citrullinated filaggrin in the first-generation anti-CCP test, in which the sensitivity increased to more than 70%.

α-Enolase, unlike filaggrin, is abundantly expressed in the synovial membrane. Several lines of evidence indicate that it is citrullinated in the joint. First, it was detected in the myeloid-like HL-60s cell line, which expresses PAD and has a similar phenotype to that of cells abundant in the joint. Second, it was detected as a synovial antigen that co-localised with staining for citrullinated proteins. The staining shown in Figure 6a suggests that only a small proportion of the antigen is citrullinated *in vivo*, which might explain why we were unable to demonstrate citrullination of α-enolase by Western blotting of immunoprecipitates from synovial cells.

Although this is the first report that citrullinated α-enolase is a common target antigen in RA, native α-enolase has previously been observed as an infrequent target antigen for several autoimmune diseases [31-34]. For example, Saulot and colleagues [34] observed that antibodies against (placental) α-enolase occurred in 25% of patients with RA and were predictive of radiological progression. They found that only 8 of the 36 patients reacting with placental α-enolase also reacted with the recombinant protein. This contrasted with 19% of patients with systemic lupus erythematosus and 15% of patients with systemic sclerosis whose serum samples reacted with both forms of α-enolase. They hypothesised that RA sera reacting with placental α-enolase, but not recombinant antigen, were recognising a post-translationally modified form of α-enolase. Although it is tempting to speculate that the modification they predicted is citrullination, the most abundant of the triplet of spots identified as α-enolase in their study migrated in two-dimensional electrophoresis at a pI of 7.0, consistent with native α-enolase. However, it is possible that the two more acidic α-enolase spots, which they attributed to phosphorylation, might in fact be citrullinated. The expression of PAD2 protein in the placenta would account for a degree of deimination either *in vivo *or during extraction. It is also consistent with the identification of the Sa antigen, also of placental origin, as citrullinated vimentin [17]. The higher frequency of anti-citrullinated α-enolase in our study than that of Saulot and colleagues might be due to the fact that our cell lysates were extensively deiminated *in vitro*. This is demonstrated by the uniform migration of deiminated enolase at a pI of 5 and by the replacement of arginine by citrulline in all the peptides listed in Table 1.

In our study, 15% of the control sera reacted with native α-enolase and also with citrullinated α-enolase, whereas reactivity with the citrullinated form alone was restricted to the patients with RA. This is, again, consistent with the results of Saulot and colleagues, assuming that the placental form of the protein was partly deiminated. In turn, this suggests that RA-specific antibodies might be driven by peptides containing one or more of the 17 potential citrulline residues in the sequence of α-enolase. Binding to non-arginine containing regions might account for the 'background', and hence the apparent loss of disease specificity seen when immunoblotting with the normal sera in our study, and the non-RA sera in that of Saulot and colleagues. One way to test this would be to examine reactivity to peptides derived from citrullinated epitopes from α-enolase. Such studies are currently in progress. If both sensitivity and specificity increases for RA it might provide another assay for diagnosis of the disease. More importantly it would provide further data to support the concept that α-enolase is a driving autoantigen in RA.

The properties of citrullinated α-enolase make it an attractive synovial antigen for driving the immune response. α-Enolase is a highly conserved, multifunctional protein that, in addition to its role in glycolysis, binds plasminogen. It is known to be upregulated by hypoxia [35] and by proinflammatory stimuli [36], both of which are features of the synovial membrane microenvironment in RA. α-Enolase is expressed during cell differentiation and is used as marker of differentiation in the grading of tumours [37]. In myeloid cells, the dominant cell type in the inflamed synovium, it is co-expressed with PAD2 and PAD4. In the present study we have shown that its distribution in the subsynovium is similar to that of citrullinated proteins and PAD [38], but we have not yet demonstrated its citrullination *in vivo*.

There is substantial similarity between human and prokaryotic α-enolases (47% identity with that from *Streptococcus pyogenes*, for example), and antibodies raised against streptococcal surface α-enolase also recognise the human enzyme [36]. Thus the presence of antibodies against uncitrullinated α-enolase, in serum of individuals without RA, might be attributable to cross-reaction with bacterial epitopes. Expression of an enzyme able to citrullinate peptidylarginine has been demonstrated in the oral organism *Porphyromonas gingivalis *[39], which provides a mechanism by which antibacterial antibodies cross-react with endogenous citrullinated proteins and initiate loss of tolerance.

## Conclusion

We have demonstrated that antibodies against citrullinated α-enolase are found in 46% of serum samples from patients with RA, and that native α-enolase is abundantly expressed in rheumatoid synovium. It is upregulated by factors such as hypoxia, which are characteristic of the rheumatoid joint, and its amino-acid sequence is highly conserved between prokaryotes and higher eukaryotes, making citrullinated α-enolase a candidate target antigen in RA; this merits further investigation.

## Abbreviations

CCP = cyclic citrullinated peptide; PAD = peptidylarginine deiminase; PBS = phosphate-buffered saline; RA = rheumatoid arthritis.

## Competing interests

The authors declare that they have no competing interests.

## Authors' contributions

AK performed immunoblotting, screened sera for antibodies, detected α-enolase in synoviocytes, and participated in study design and drafting of the manuscript. VT performed the initial Western blotting, cellular fractionation and two-dimensional electrophoresis experiments. DP, KL and PD performed the immunohistochemistry. DM participated in the study design and established the cell culture methodology. PCT performed the synovial biopsies and participated in study design and drafting of the manuscript. RW was responsible for mass spectrometric characterisation of citrullinated α-enolase, participated in the study design and helped to draft and edit the manuscript. PJV conceived of the study, participated in its design and edited the manuscript. All authors read and approved the final version.

## Figures and Tables

**Table 1:** Peptides from deiminated α-enolase sequenced by tandem mass spectrometry

*m*/*z *(charge)	Location	Matched sequence
401.24 (2+)	221–227	EGLELLK
480.77 (2+)	81–88	LNVTEQEK
674.34 (2+)	394–405	TGAPC**(Cit)**SE**(Cit)**LAK
696.87 (2+)	422–433	FAG**(Cit)**NF**(Cit)**NPLAK
817.41 (2+)	343–357	VNQIGSVTESLQACK
837.38 (3+)	285–385	DYPVVSIEDPFDQDDWGAWQK
846.95 (2+)	406–419	YNQLL**(Cit)**IEEELGSK
980.98 (2+)	202–220	DATNVGDEGGFAPNILENK
878.45 (3+)	5–27	IHA**(Cit)**EEIFDS**(Cit)**GNPTVEVDLFTSK
1,177.10 (2+)	372–393	SGETEDTFIADLVVGLCTGQIK
915.14 (3+)	202–227	DATNVGDEGGFAPNILENKEGLELLK
988.15 (3+)	256–280	YDLDFKSPDDPS**(Cit)**YISPDQLADLYK
1,017.04 (2+)	306–325	FTASAGIQVVGDDLTVTNPK
925.24 (4+)	126–161	GVPLY**(Cit)**HIADLAGNSEVILPVPAFNVINGGSHAGNK

Cit, citrulline.

**Table 2:** Peptides from non-citrullinated α-enolase sequenced by tandem mass spectrometry

*m*/*z *(charge)	Location	Matched sequence
401.24 (2+)	221–227	EGLELLK
403.73 (2+)	406–411	YNQLLR
452.75 (2+)	412–419	IEEELGSK
480.77 (2+)	81–88	LNVTEQEK
572.30 (2+)	183–192	IGAEVYHNLK
703.86 (2+)	15–27	GNPTVEVDLFTSK
713.34 (2+)	269–280	YISPDQADLYK
482.29 (3+)	80–91	KLNVTEQEKIDK
817.41 (2+)	343–357	VNQIGSVTESLQACK
785.09 (3+)	372–393	SGETEDTFIADLVVGLCTGQIK
915.14 (3+)	202–227	DATNVGDEGGFAPNILENKEGLELLK
753.66 (4+)	132–161	HIADLAGNSEVILPVPAFNVINGGSHAGNK
